# Elevation of hypothalamic ketone bodies induces a decrease in energy expenditures and an increase risk of metabolic disorder

**DOI:** 10.1016/j.molmet.2024.101926

**Published:** 2024-03-28

**Authors:** Lionel Carneiro, Rocco Bernasconi, Adriano Bernini, Cendrine Repond, Luc Pellerin

**Affiliations:** 1 Department of Physiology, University of Lausanne, 1005 Lausanne, Switzerland; 2 University and CHU of Poitiers, INSERM U1313, Poitiers, France

**Keywords:** Obesity, Metabolism, Ketone bodies, Neuroscience

## Abstract

**Objective:**

Ketone bodies (such as β-hydroxybutyrate or BHB) have been recently proposed as signals involved in brain regulation of energy homeostasis and obesity development. However, the precise role of ketone bodies sensing by the brain, and its impact on metabolic disorder development remains unclear. Nevertheless, partial deletion of the ubiquitous ketone bodies transporter MCT1 in mice (HE mice) results in diet-induced obesity resistance, while there is no alteration under normal chow diet. These results suggest that ketone bodies produced during the high fat diet would be important signals involved in obesity onset.

**Methods:**

In the present study we used a specific BHB infusion of the hypothalamus and analyzed the energy homeostasis of WT or HE mice fed a normal chow diet.

**Results:**

Our results indicate that high BHB levels sensed by the hypothalamus disrupt the brain regulation of energy homeostasis. This brain control dysregulation leads to peripheral alterations of energy expenditure mechanisms.

**Conclusions:**

Altogether, the changes induced by high ketone bodies levels sensed by the brain increase the risk of obesity onset in mice.

## Highlights

• High ketone bodies levels sensed by the hypothalamus are deleterious.
• Ketone bodies sensing by the hypothalamus regulates energy expenditures.
• Decreasing hypothalamic ketone bodies sensing might protect against diet-induced obesity.

## 1 Introduction

Energy homeostasis results from a complex communication between organs and tissues of the body. This communication is tightly regulated and allows the maintenance of a constant body weight and internal parameters such as temperature or blood glucose levels. The brain plays a key role in this complex regulatory system by constantly sensing and integrating various signals from the periphery informing it about the energetic status of the body [1]. Indeed, once it has integrated information received from the periphery, the brain sends back an adaptive response to adjust the function of organs involved in energy homeostasis [2]. The brain also modulates food intake and energy supply for the entire body [2]. Within the brain, the hypothalamus has an important role in this regulatory loop. Indeed, the hypothalamus is described as fully equipped for the detection of nutrients or hormonal signals from the periphery [3]. Therefore, the hypothalamic detection of nutrients (e.g. glucose and fatty acids) and hormones (e.g. leptin, ghrelin and insulin) will modulate both energy expenditure and energy supply of the body [4]. More recently, Ketone Bodies (KB) have been shown to be involved in the cerebral control of energy homeostasis. As demonstrated previously, increased levels of circulating KB are sensed by the hypothalamus and trigger an adaptive food intake response [[5], [6], [7], [8], [9], [10]]. However, discrepancies between results suggest that the mechanisms and roles are yet to be completely understood. Among others, strategies (KB infusion or High Fat Diet (HFD)), time course (hours vs days) and models (mice vs rats) could in part account for the differences observed. Notwithstanding, all results suggest a role in obesity development. Furthermore, a mouse model haploinsufficient for the ubiquitous MCT1 transporter (Monocarboxylate Transporter 1, HE mice), the main KB transporter, displays a resistance to HFD-induced obesity [11]. Under normal chow diet though, HE mice do not show any significant phenotypic difference when compared to wildtype (WT) mice. These results suggest that a specific signal produced during HFD and requiring MCT1 is involved in obesity development.

Blood KB levels are low under normal conditions but increase during HFD [12,13]. In parallel, an increase of MCTs expression is observed in the brain during HFD [14]. These data suggest a role of KB as signals involved in obesity development as well as in brain control of energy homeostasis. MCT1 was identified as the main KB transporter in the central nervous system. Although it can be expressed by different brain cell types (e.g. astrocytes, endothelial cells, microglia, tanycytes) [15], it was found to be present on specific hypothalamic neuronal populations [16] including NPY neurons [5,17]. Thus, KB levels could potentially modulate the participation of such hypothalamic neurons in energy homeostatic mechanisms. Interestingly, opposite actions could result from a biphasic effect of KB. Indeed, physiological hyperketonemia occurs during fasting periods, which is associated with food intake stimulation [18,19]. In accordance with such a hypothesis, a KB infusion of 24 h produces such a biphasic effect [6]. This result suggests that acute and chronic hyperketonemia could have a distinct physiological and dysregulated signaling effect, respectively.

Therefore, in the present study, we aimed to address the impact of a chronic hypothalamic increase of KB levels on the hypothalamic control of energy homeostasis. β-hydroxybutyrate (BHB), the main physiological KB, was infused specifically and directly into the hypothalamus for 7 days in both WT and HE mice. It was observed that a chronic hyperketonemia sensed by the hypothalamus in WT mice induces a decrease in energy expenditure. In parallel, subcutaneous white adipose tissue (scWAT) underwent a shift towards a beige phenotype. Altogether, these results reveal a phenotype with an increased risk of obesity development in WT mice. On the other hand, HE mice displayed opposite results with increased energy expenditure and a phenotype of obesity resistance.

## 2 Materials and methods

### 2.1 Animals

MCT1+/+ (WT) and MCT1+/− (Heterozygote (HE)) mice were bred at the University of Lausanne animal facility and 8 weeks male littermates individually housed in a controlled environment (12-h light–dark cycle, lights on at 7 AM, 22 °C) with ad libitum access to food (Kliba Nafag standard diet no. 3336; Kliba Nafag, Kaiseraugst, Switzerland) and water were used for all experiments. Mice were genotyped as described previously [11]. BHB perfusion was performed using osmotic pumps model 2006 (Alzet, Cupertino, CA, USA) at a rate of 0.15 μL/h for 7 days. BHB was infused within the hypothalamus area by connecting the osmotic pump to a brain infusion kit 3 (Alzet) implanted at the following coordinates according to the Stereotaxic Atlas (The mouse brain in stereotaxic coordinates, K.B.J. Franklin, G. Paxinos, 1997): AP coordinate: 1.5 mm; DV coordinate: 5.75 mm; ML 0.1 mm. The osmotic pump was subcutaneously inserted in the side of the mouse and connected to the Alzet Cannula by a subcutaneous catheter of 3 cm filled with saline solution. The control group (WT-CSF or HE-CSF groups) received aCSF (NaCl at 0.3 M, KCl at 6 mM, CaCl_2_ ⋅ 2H_2_O at 2.8 mM, MgCl_2_ ⋅ 6H_2_O at 1.6 mM, Na_2_HPO_4_ ⋅ 7H_2_O at 1.6 mM and NaH_2_PO_4_ ⋅ H_2_O at 0.39 mM) whereas the ketone bodies infused groups (WT-BHB or HE-BHB) received a solution of 200 mg/mL aCSF diluted BHB (DL-β-hydroxybutyric acid, Sigma–Aldrich Chemie GmbH, Steinheim, Germany). The dose chosen was calculated based on a volume of mouse hypothalamus of 5 μL such that the concentration of BHB reaching this area would be approximately 6 mM. This concentration corresponds to a similar hyperketonemia induced by a fast. Both solutions have an adjusted pH between 7.3 and 7.4. During the 7 days of infusion food intake was monitored daily using the Phenomaster system (TSE, Germany). At the end of the 7 days infusion period, body composition was determined using an EchoMRI (EchoMRI LLC, USA), then mice were anesthetized using pentobarbital sodium (50 mg/kg) and tissues harvested, snapped frozen and kept at −80 °C before analysis. All procedures involving mice followed the European Communities Council Directive (86/609/EEC) and were approved by the Service de la Consommation et des Affaires Vétérinaires du Canton de Vaud (Authorization N° VD2634).

### 2.2 Blood analysis

Ketonemia (Free Style precision, Abbott, Oxon, UK), glycemia (Benecheck plus multi-monitoring system, General Life Biotechnology Co., Taiwan) and lactatemia (The Edge analyser, Apex Biotechnology Corp., Taiwan) were measured at the end of the 7 days infusion. Insulinemia (Rat/mouse Insulin ELISA Kit, Millipore, Billerica MA, USA) and plasma leptin (Rat/mouse Leptin ELISA Kit, Millipore, Billerica MA, USA) were also measured using the indicated Elisa kit. Catecholamines concentrations were determined using an Elisa kit (Rocky Mountain Diagnostics Inc., USA).

### 2.3 Indirect calorimetry

Mouse energy expenditures were measured in metabolic cages using the Oxymax apparatus (Columbus instruments, USA). Mice were weighed and placed in metabolic cages during a night, to adapt to individual cages with free access to water and food. CO_2_ production (VCO_2_) and O_2_ consumption (VO_2_) and activity (XAMB and XTOT) of mice were measured during the 48 h following the habituation period. Respiratory exchange ratio (RER) was determined by the ratio VCO_2_/VO_2_. Metabolic rate was calculated using the Weir equation (Metabolic rate (kcal per day) = 1440 (3.9 VO_2_ + 1.1 VCO_2_)). Activity determination corresponds to mouse displacements since it corresponds to the number of movements after at least 4 consecutive movements.

### 2.4 Temperature monitoring and cold tolerance test

An implantable temperature sensor was inserted intraperitoneally during the stereotaxic surgery. The temperature was then recorded every 15min during the 7 days of infusion using the Anipill system (Animals monitoring, France). For cold tolerance tests, after a 6 h fast, mice were placed at 4 °C and temperature was recorded every 5min during 4 h.

### 2.5 Protein extraction and Western blots

Frozen tissues were homogenized in RIPA lysis buffer (Millipore, Zug, Switzerland) with phosphatase and protease inhibitors (Pierce, Lausanne, Switzerland). Thirty mg of total proteins were loaded on SDS-PAGE gels and transferred to nitrocellulose membranes. Membranes were blocked with TBS containing 0.1% Tween 20 and 5% BSA, then incubated overnight with the primary antibody directed against UCP1 (dilution 1/1000) (Cell Signaling, USA) or Vinculin (dilution 1/750) (Sigma–Aldrich Chemie GmbH, Steinheim, Germany). Blots were revealed by chemiluminescence (WesternBright ECL; Witec ag, Luzern, Switzerland) and imaged with a detection system (Chemidoc XRSþ, Biorad, Switzerland). Densitometric analysis of chemiluminescent signals was performed using Image lab software and the densities ratio of UCP1/Vinculin used to normalize the results.

### 2.6 RNA extraction, reverse transcription and quantitative real time PCR

Tissue samples collected were lysed and homogenized in 350 μl of lysis buffer (RLT Buffer, Qiagen) using the Fast prep 24 lyzer (MPbio, Luzern, Switzerland) according to manufacturer's instructions. Total RNA was isolated on spin columns with silica-based membranes (RNeasy Mini Kit, Qiagen, Basel, Switzerland), following manufacturer's instructions. RNA was eluted with 30 μl of H_2_O. Two hundred ng of purified RNA was reverse transcribed in a volume of 50 μl using the RT High-Capacity RNA-to-cDNA Kit (Applied Biosystems, Rotkreuz, Switzerland). Quantitative real-time PCR analysis was performed on cDNA obtained with the Applied Biosystems 7900 (Applied Biosystems, Rotkreuz, Switzerland) Real-Time PCR System using Power SYBR Green Taq polymerase master mix (Applied Biosystems, Rotkreuz, Switzerland). Primer sequences used for mRNA quantification are listed in Table 1. Data were analyzed with RQManager 1.2 software (Applied Biosystems, Rotkreuz, Switzerland) for relative quantitation of gene expression (RQ) using the 2−ΔΔCT method with *PolR2α* used as housekeeping gene.

**Table 1:** Primer sequences list.

	Forward	Reverse
**AgRP**	ATG CTG ACT GCA ATG TTG CTG	CAG ACT TAG ACC TGG GAA CTC C
**Alpha 1 adrenergic Recptor**	AGTGGGTGTCTTCCTAGCC	GCCTAGAACCTCCATAGTGGC
**Alpha 2 adrenergic Recptor**	CTTTTGCACGTCGTCCATAGT	CGGTGACAATGATGGCCTTGA
**Atp5b**	CACAATGCAGGAAAGGATCA	GGTCATCAGCAGGCACATAG
**Atp6v0d2**	ACTTTTGGTGTTGTTCTGGGAA	GCATGAACAGGATCTCAGGC
**Atp9b**	TCTGGTAGTGTCCTGCTCACAG	TCGTAACGGCCAAAACAAAT
**Beta 1 adrenergic Recptor**	AGCCCTTGGTGGAGTTCTAC	CCCCTCGGAGGTTCTGACA
**Beta 2 adrenergic Recptor**	AGGAGGGTTTGGGGAAGTTTA	CATGATCCTCTCGTTCAAAGCC
**Beta 3 adrenergic Recptor**	TCTCTGGCTTTGTGGTCGGA	GTTGGTTATGGTCTGTAGTCTCG
**BHBDH**	TGC AAC AGT GAA GAG GTG GAG AAG	CAA ACG TTG AGA TGC CTG CGT TGT
**CART**	CCC GAG CCC TGG ACA TCT A	GCT TCG ATC TGC AAC ATA GCG
**CHREB**	CAA GTT GCT ATG CCG GGA CAA	CCT CCG TTG CAC ATA CTG ATT
**Cidea**	TGC TCT TCT GTA TCG CCC AGT	GCC GTG TTA AGG AAT CTG CTG
**COX1**	CCA GAG TCA TGA GTC GAA GGA	CCA GGT CCA GAT CTC AGG GA
**Cox2**	GCCGACTAAATCAAGCAACA	CAATGGGCATAAAGCTATGG
**Cox4**	GCACATGGGAGTGTTGTGA	CCTTCTCCTTCTCCTTCAGC
**Cox5α**	GGGTCACACGAGACAGATGA	GGAACCAGATCATAGCCAACA
**Cytb**	CATTTATTATCGCGGCCCTA	TGTTGGGTTGTTTGATCCTG
**Dio2**	CAGTGTGGTGCACGTCTCCAATC	TGAACCAAAGTTGACCACCAG
**Elovl3**	TCC GCG TTC TCA TGT AGG TCT	GGA CCT GAT GCA ACC CTA TGA
**Err-α**	GCAGGGCAGTGGGAAGCTA	CCTCTTGAAGAAGGCTTTGCA
**GLUT1**	GCC CCC AGA AGG TTA TTG A	CGT GGT GAG TGT GGT GGA TG
**GLUT2**	TTC CGG AAG AAG AGT GGT TCG	TGG TCG GTT CCT CGG TTT TAG
**GLUT3**	CGT CTG CCA AAG CGG TTG ACA	TGC TGA CTG CCC TCT GGT CCT TAT G
**GLUT4**	ACA CTG GTC CTA GCT GTA TTC T	CCA GCC ACG TTG CAT TGT A
**HMGcs2**	TGG TTC AAG ACA GGG ACA CAG AAC	AGA GGA ATA CCA GGG CCC AAC AAT
**MCT1**	TTG GAC CCC AGA GGT TCT CC	AGG CGG CCT AAA AGT GGT G
**MCT2**	CTC TGG CTG GTA AAT TGC TTG A	ACG ACT GTT CCG CTG GCT AT
**MCT4**	GTG TCG CTG TAG CCA ATC CC	GGC TGT TTT ATC ATC ACG GGT T
**NPY**	ATG CTA GGT AAC AAG CGA ATG G	TGT CGC AGA GCG GAG TAG TAT
**Nrf1**	GAACTGCCAACCACAGTCAC	TTTGTTCCACCTCTCCATCA
**Pgc1α-total**	TGATGTGAATGACTTGGATACAGACA	GCTCATTGTTGTACTGGTTGGATATG
**PolR2α**	CCC TCA TCA TAC CTG GAC ACA TC	GTA AGG GCC ACT ATC TTC ATC ATC
**POMC**	ATG CCG AGA TTC TGC TAC AGT	TCC AGC GAG AGG TCG AGT TT
**PRDM16**	CAG CAC GGT GAA GCC ATT C	GCG TGC ATC CGC TTG TG
**Slit2**	GATTCTGGTGCACTTGTGCTG	TGTGTATTCCGGTGGGCAAA
**Uqcrb**	AGGCTTCCTGAGGACCTTTA	TCCTTAGGCAAGATCTGATGC

### 2.7 Statistical analysis

Results are presented as mean ± SEM. Statistical analysis was performed using GraphPad-Prism 6.01. Normality was tested with the Kolmogorov–Smirnov test. ANOVA analyses were performed to compare the four groups and significant differences are presented as ∗, ∗∗, ∗∗∗ or ∗∗∗∗ on graphics and histograms for p values < 0.05, 0.01, 0.001, or 0.0001 respectively.

## 3 Results

### 3.1 Ketone bodies infusion for seven days into the hypothalamus induces an increased weight gain in WT mice

**Figure 1:**
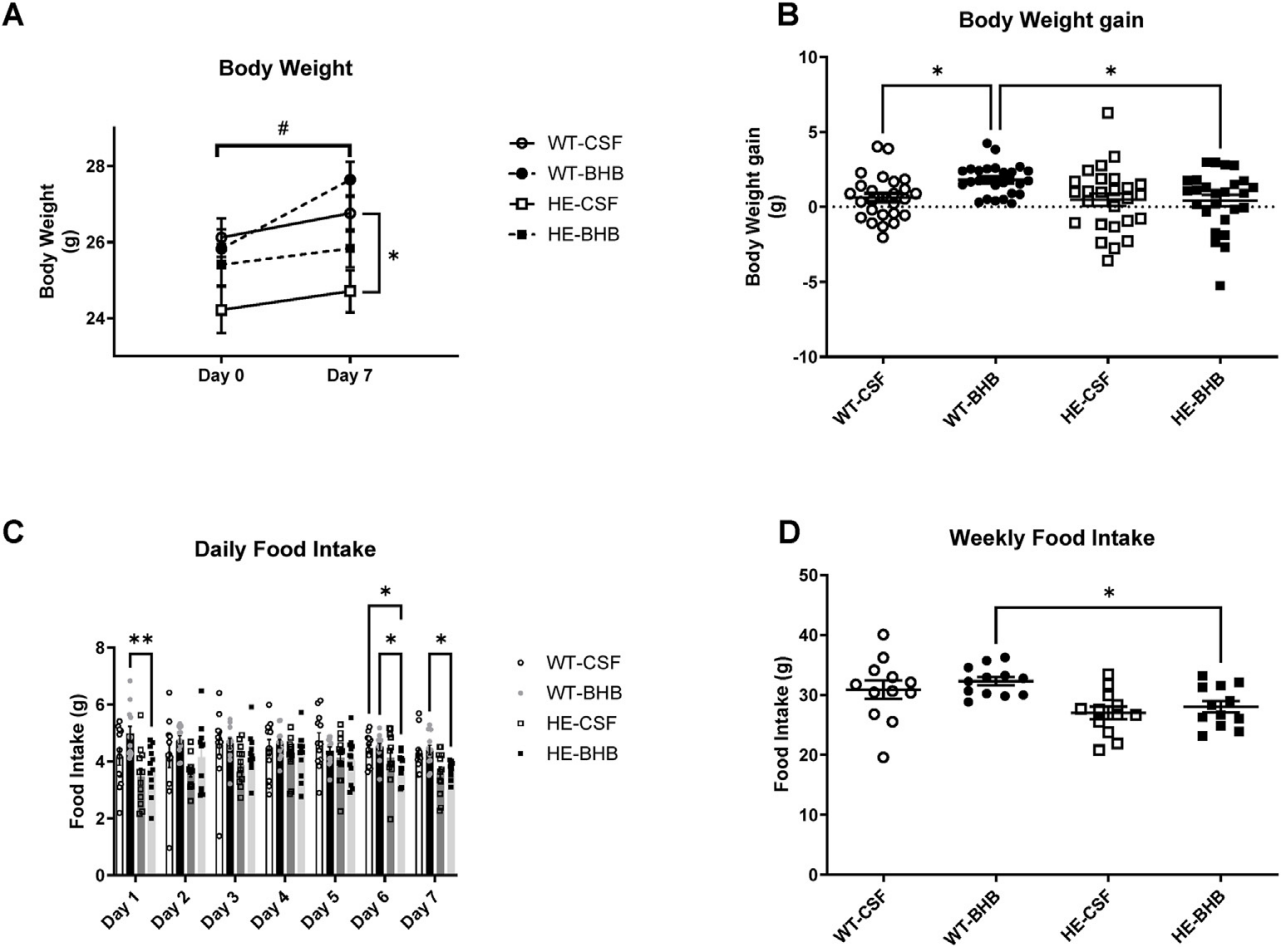
**Chronic hypothalamic β-hydroxybutyrate infusion increases body weight only in MCT1+/+ mice.** Body weight and food intake monitoring of mice receiving a 7-days β-hydroxybutyrate infusion to the hypothalamus. (A) Body weight (B) Body weight gain (C) Daily food ingested and (D) 7-days food intake. N = 26 for A and B, n = 12 for C and D, Statistical differences are represented with ∗ when p < 0.05, ∗∗ when p < 0.01, ∗∗∗ when p < 0.001 and ∗∗∗∗ when p < 0.0001. In panel A, # indicates a statistical difference of p < 0.05 between day 0 and 7 for the WT-BHB group.

Ketogenic diets are used classically to induce weight loss by stimulating lipid catabolism. Recent research indicates that elevated brain KB alters food intake regulation [[5], [6], [7], [8], [9], [10]]. Therefore, we first assessed food intake and body weight of WT mice during a 7-day hypothalamic infusion of either artificial CerebroSpinal Fluid (aCSF) (WT-CSF) or BHB (WT-BHB). At day 7, only WT-BHB mice show a significantly higher body weight compared to day 0 (Figure 1A). Furthermore, weight gain during the 7 days is significantly higher in WT-BHB mice compared to the WT-CSF control group (Figure 1B). In parallel, the daily measurement of food intake shows an increase in WT-BHB mice the first 24 h compared to WT-CSF group. However, in the following 6 days, there is no difference in food ingested between groups (Figure 1C). Consequently, the total food ingested during the 7 days infusion remains similar in both WT-BHB and WT-CSF mice (Figure 1D).

**Figure 2:**
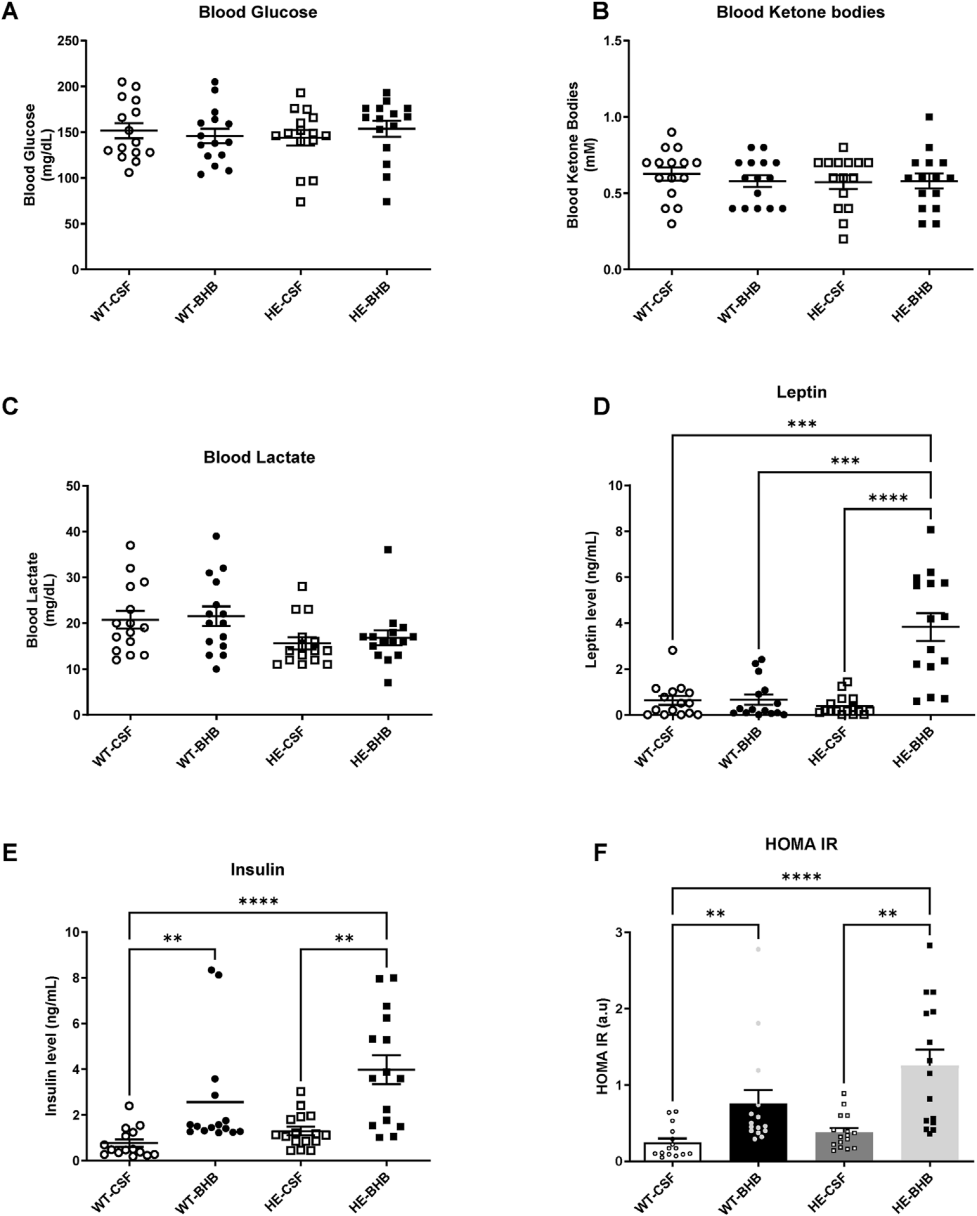
**Chronic hypothalamic β-hydroxybutyrate infusion induces changes in endocrine responses.** Blood analyses include (A) Glucose (B) Ketone Bodies (C) Lactate (D) Leptin and (E) Insulin levels as well as (F) The calculated Homa IR. n = 15. Statistical differences are represented with ∗ when p < 0.05, ∗∗ when p < 0.01, ∗∗∗ when p < 0.001 and ∗∗∗∗ when p < 0.0001.

**Figure 3:**
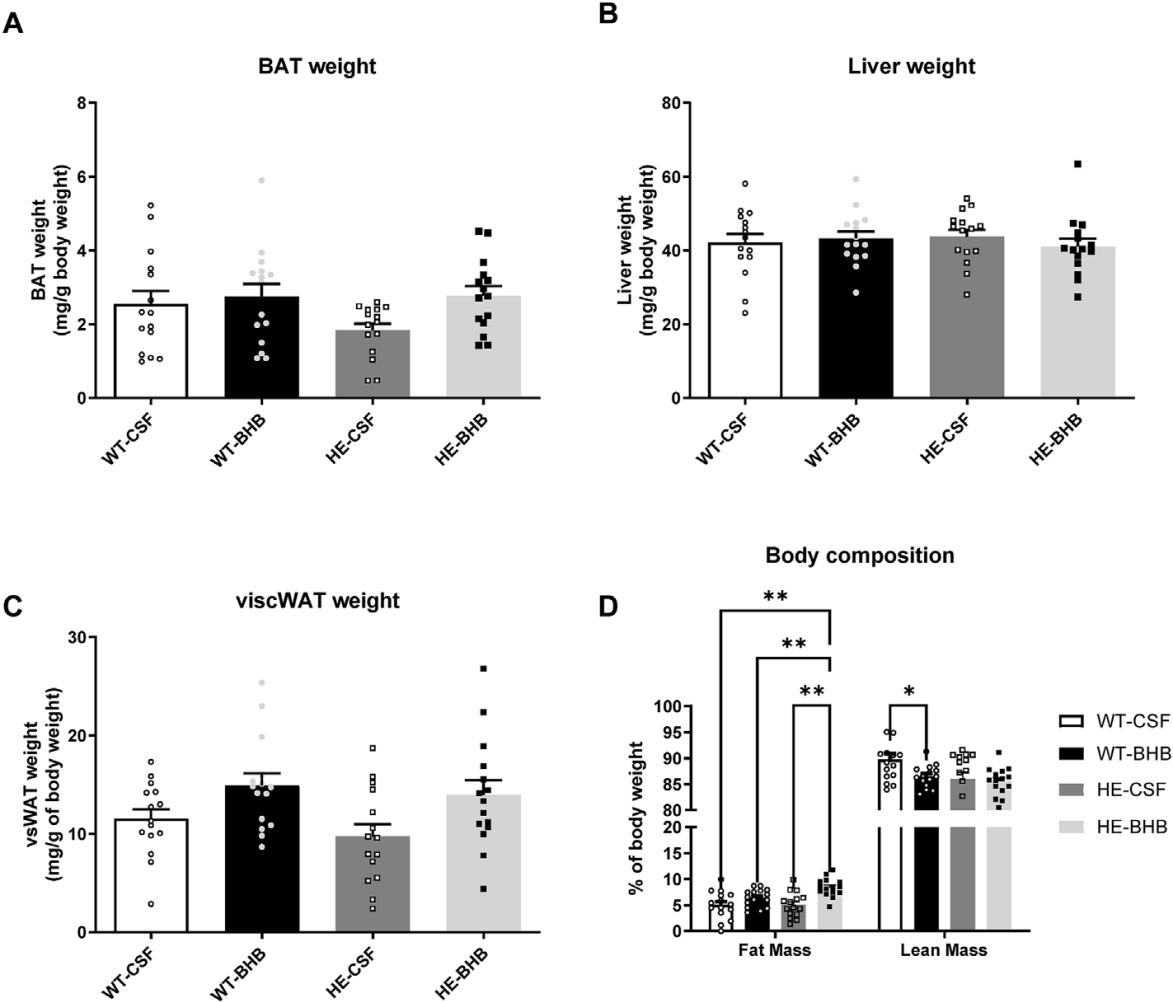
**Chronic hypothalamic β-hydroxybutyrate infusion alters body composition.** Body composition was assessed by determining the weight of (A) Brown Adipose tissue (BAT), (B) Liver and (C) visceral White Adipose tissue (vsWAT), as well as performing (D) echoMRI body composition analysis. N = 26 for A and B, n = 12 for C and D Statistical differences are represented with ∗ when p < 0.05, ∗∗ when p < 0.01, ∗∗∗ when p < 0.001 and ∗∗∗∗ when p < 0.0001.

**Figure 4:**
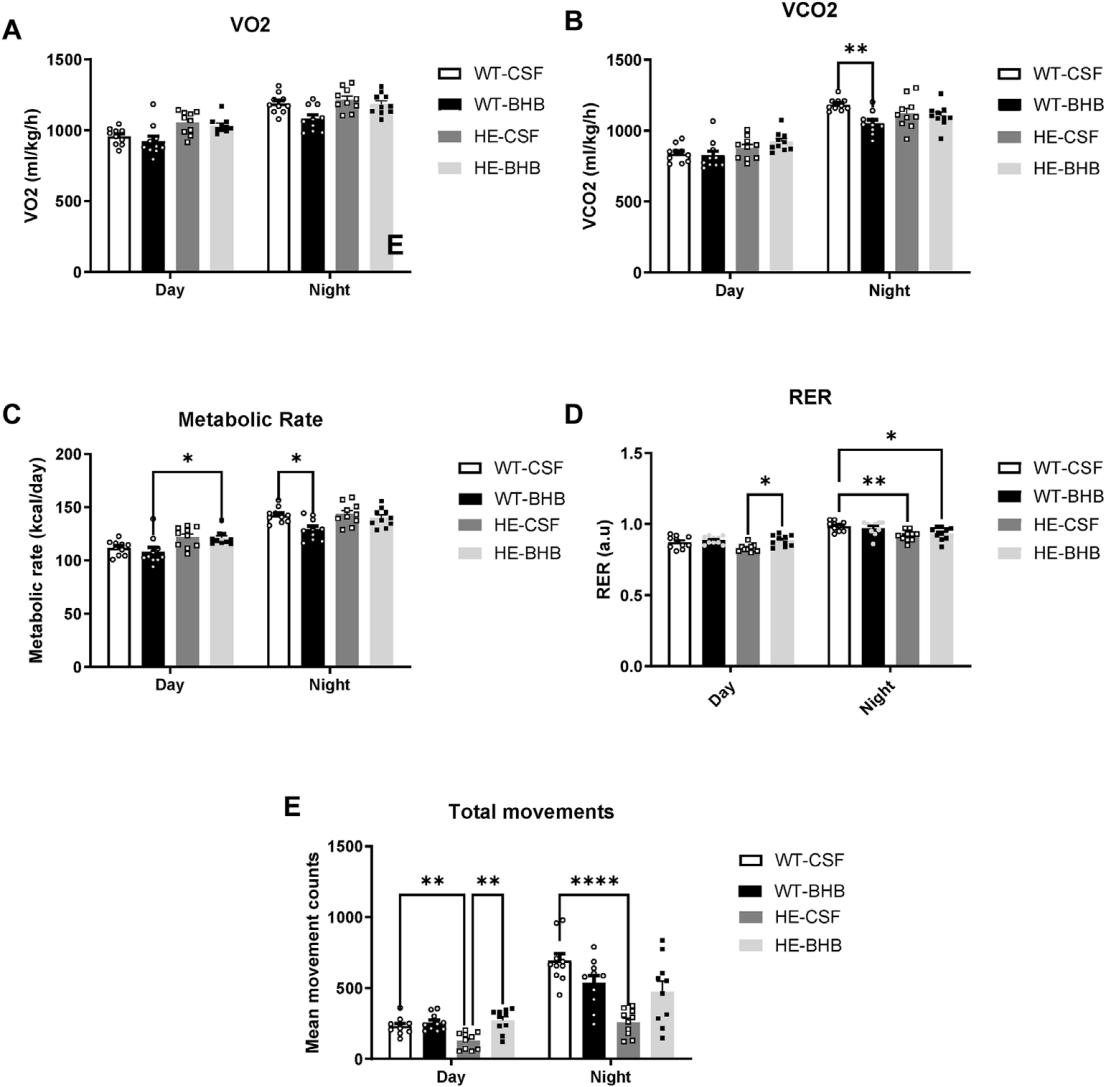
**Chronic hypothalamic β-hydroxybutyrate infusion alters energy expenditures.** Energy expenditures were determined via indirect calorimetry experiments and the determination of (A) VO2, and (B) VCO2 utilization/release while the metabolic rate was calculated using the Weir equation (C). The calculated Respiratory Exchange Ratio (RER) (VCO2/VO2 ratio) (D) and total movements recorded (E) were also evaluated. n = 10 for each group. Statistical differences are represented with ∗ when p < 0.05, ∗∗ when p < 0.01, ∗∗∗ when p < 0.001 and ∗∗∗∗ when p < 0.0001.

Blood analysis of glucose, lactate and leptin did not show any differences between groups after 7 days of infusion (Figure 2). KB blood levels remain also unchanged indicating that BHB infused in the brain did not alter the whole body ketonemia (Figure 2B). Therefore, only the hypothalamus had been stimulated. However, blood insulin shows a threefold increase in WT-BHB mice (Figure 2E). Together with the unchanged blood glucose, the hyperinsulinemia leads to the increased calculated Homa-IR, indicating an insulin resistance in WT-BHB mice (Figure 2F). Furthermore, no alteration of Brown Adipose Tissue (BAT), Liver or visceral White Adipose Tissue weight was observed (Figure 3A–C). Accordingly, no change in fat mass was measured by echoMRI (Figure 3D). The lean mass though was significantly decreased in WT-BHB (Figure 3D). Such a decrease is consistent with decreased energy expenditure measured by indirect calorimetry (Figure 4A–E). Indeed, VCO_2_ presented a decrease that is accompanied by reduced activity, all these changes occurring specifically during the active (dark) phase (Figure 4A–E). Accordingly, the metabolic rate is decreased during the dark phase indicating decreased energy expenditure (Figure 4C). Finally, the respiratory exchange ratio (RER) was unaffected indicating that no change in nutrient oxidation occurred (Figure 4D).

### 3.2 Chronic hyperketonemia in the hypothalamus does not alter orexigenic neuropeptides nor nutrient transporters expression in WT mice

**Figure 5:**
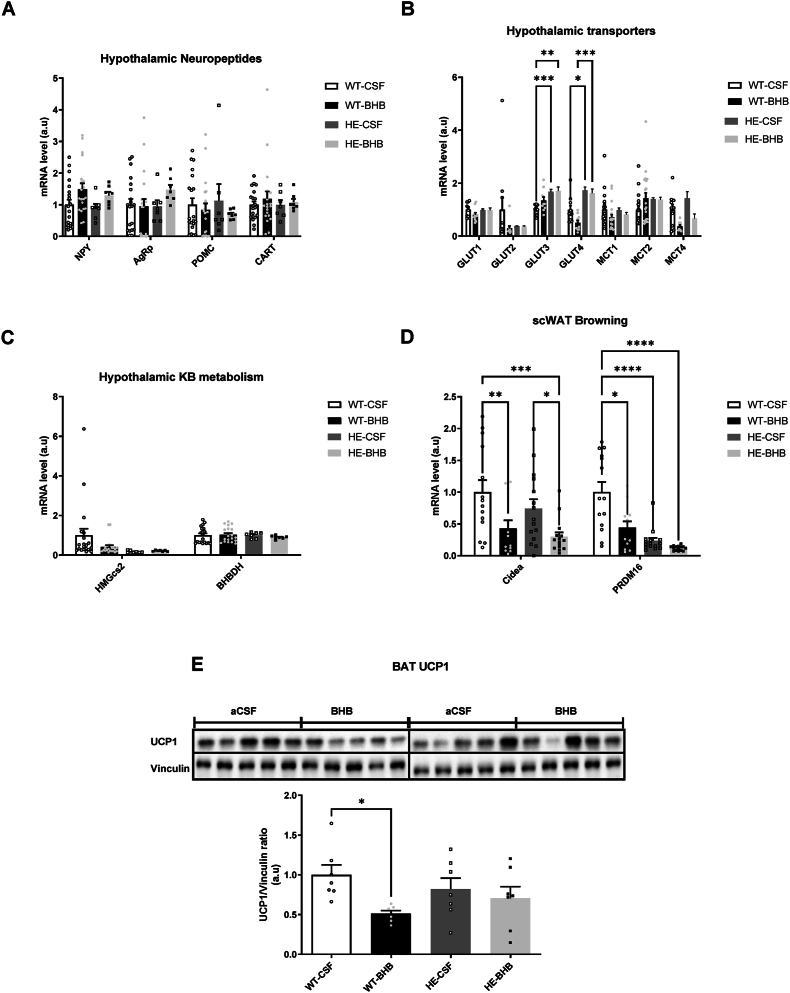
**Hypothalamic ketone bodies sensing is responsible for thermogenesis program adaptations.** β-hydroxybutyrate administered within the hypothalamus is responsible for changes in hypothalamic gene expression of (A) Neuropeptides involved in food intake control and (B) Glucose and monocarboxylate transporters while (C) Genes involved in ketone bodies metabolism are not affected. Furthermore, thermogenesis regulation was assessed by the analysis of (D) Genes involved in the browning process in sub-cutaneous WAT as well as (E) UCP1 protein levels in BAT. N = 24 in panels A, B and C; n = 14 for WT-CSF and n = 12 for WT-BHB in panel D and n = 7 in panel E. Statistical differences are represented with ∗ when p < 0.05, ∗∗ when p < 0.01, ∗∗∗ when p < 0.001 and ∗∗∗∗ when p < 0.0001.

To assess the neuronal network involved in hypothalamic energy homeostasis regulation upon chronic hyperketonemia, we analyzed mRNA levels of the melanocortinergic system. Results show that both orexigenic NPY and CART or anorexigenic neuropeptides POMC and CART mRNAs were unaffected (Figure 5A). These neurons are activated or silenced depending on the energy availability signaled through nutrient sensing mechanisms involving glucose metabolism and direct sensing of KB. Therefore, we analyzed the levels of transporter mRNA expression for these different nutrients. Again, no change in mRNA expression was measured in WT-BHB mice (Figure 5B). Finally, despite the increase in BHB within the hypothalamus, we did not observe any change in mRNA levels for genes coding for enzymes involved in KB synthesis (HMGcs2) or utilization (BHBDH) (Figure 5C).

### 3.3 Hypothalamic infusion of BHB for 7 days in MCT1+/+ mice induces specific alterations in sub-cutaneous WAT gene expression

Hypothalamic nutrient sensing is involved in the adaptive response that leads to changes in peripheral tissue function. Therefore, we investigated putative metabolic reprogramming of the Liver, visceral and sub-cutaneous White Adipose Tissue (vsWAT and scWAT), and Brown Adipose Tissue (BAT). Results indicate that Liver and vsWAT do not show any gene expression changes in WT-BHB mice. However, BAT displayed a decrease in protein UCP1 levels in WT-BHB mice suggesting changes in thermoregulation mechanisms (Figure 5E). Interestingly, Cidea and PRDM16 mRNA levels were also decreased in scWAT (Figure 5D). Such decreases have been previously associated with a reduction in the browning of WAT, and thus support the altered energy expenditures and thermogenesis observed here.

### 3.4 Cold tolerance and temperature regulation are unchanged by a chronic hypothalamic perfusion of BHB in WT mice

**Figure 6:**
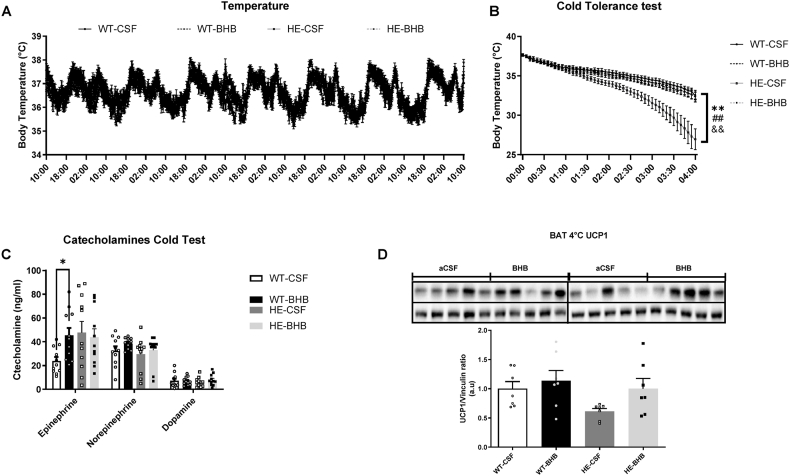
**Long term hyperketonemia sensed in the hypothalamus favors thermogenesis during a cold exposure.** During the 7 days of infusion (A) Daily body temperature was unchanged in mice compared to controls. Both groups also display a similar tolerance to cold (B). However, β-hydroxybutyrate infused mice need higher epinephrine levels to maintain their body temperature during cold tolerance (C). Cold exposure did not modify UCP1 protein levels between WT-CSF and WT-BHB mice. However, it raised UCP1 protein levels in HE-BHB mice vs. HE-CSF mice. n = 12 for each group for A and B and n = 11 in each group for panel C, and n = 7 for each group for panel D. Statistical differences are represented with ∗∗ when p < 0.01 between WT-CSF and HE-CSF, ## when p < 0.01 between WT-BHB and HE-CSF and && when p < 0.001 between HE-CSF and HE-BHB.

**Figure 7:**
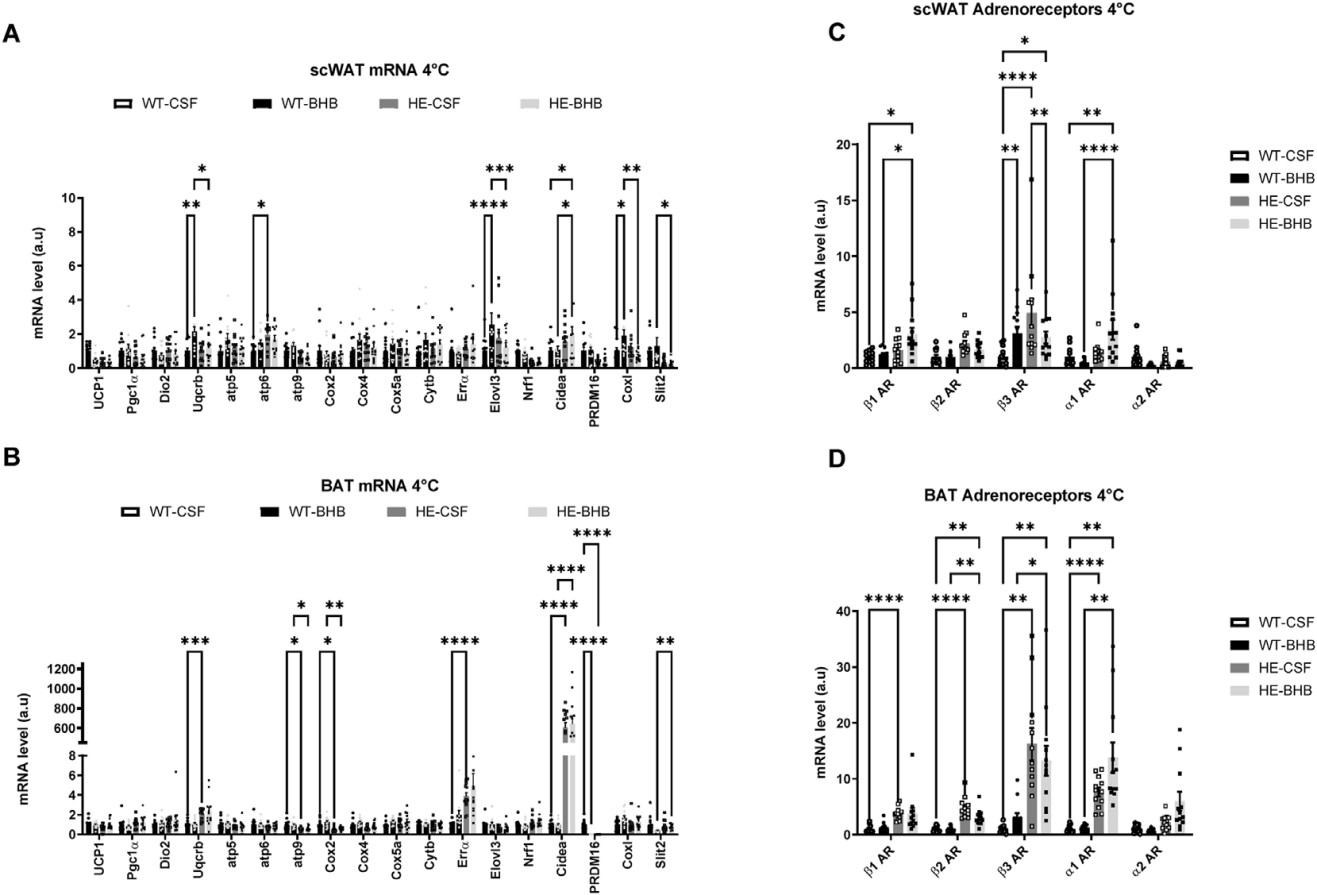
Mice receiving β-hydroxybutyrate within the hypothalamus for 7 days and exposed to cold overexpress genes involved in mitochondrial function and lipid storage. Mice subjected to a 4 °C cold exposure display gene expression alterations in adipose tissue when receiving β-hydroxybutyrate. (A) subcutaneous White Adipose tissue (scWAT) and (B) Brown Adipose tissue (BAT) expression levels of genes coding for enzymes involved in cold tolerance are presented. (C) scWAT and (D) BAT expression levels of genes coding for adrenoceptors. n = 12 for each group and each gene analyzed. Statistical differences are represented with ∗ when p < 0.05, ∗∗ when p < 0.01, ∗∗∗ when p < 0.001 and ∗∗∗∗ when p < 0.0001.

Previous results suggested that thermoregulation could be affected by chronic hypothalamic hyperketonemia. Therefore, temperature regulation was measured using an inserted telemetric temperature controller during the 7 days of infusion. No difference was observed between WT-CSF and WT-BHB groups maintained at 22 °C (Figure 6A). To probe the thermoregulation system, we measured the response of mice to a 4 °C cold exposure after the 7 days infusion of BHB. We observed that the temperature change after cold exposition is similar in WT-CSF and WT-BHB mice (Figure 6B). However, epinephrine levels are increased by cold exposure in WT-BHB mice. This result indicates that the thermogenic mechanisms have been activated by the treatment (Figure 6C) although no alteration in UCP1 protein levels was observed (Figure 6D). mRNA analysis in scWAT and BAT following cold exposure revealed an increased expression of genes involved in mitochondriogenesis in WT-BHB mice supporting an increased thermogenesis (Figure 7A,B). In addition, scWAT also showed increased expression of the β3-adrenoceptor mRNA (Figure 7C). Finally, BAT adrenoceptors mRNA levels remain unchanged. These alterations of adrenergic receptors suggest changes in adrenergic signaling which could be due to changes in nervous efferent signals.

### 3.5 HE mice infused for 7 days into the hypothalamus with BHB do not show weight gain alteration

Haploinsufficient MCT1 mice resist to diet-induced obesity while they exhibit no phenotypic alteration under normal chow. We thus hypothesized that a signal produced during HFD exposure requiring MCT1 could be involved in obesity onset. Therefore, we tested the effect of BHB infusion into the hypothalamus during 7 days in HE mice. Contrary to WT-BHB mice, HE-BHB mice did not show body weight differences compared to HE-CSF mice when infused with BHB into the hypothalamus (Figure 1A,B). In fact, HE mice infused with BHB showed a lower weight gain than MCT1+/+ mice when infused with BHB in the hypothalamus (Figure 1B). Food intake measurements also showed a similar food intake both on a daily basis and over the 7 days period between HE-CSF and HE-BHB mice (Figure 1C,D). In accordance with weight gain, HE mice display a lower food intake compared to WT mice when infused with BHB especially at days 6 and 7 (Figure 1C). This decrease results in a decreased food intake during the 7 days infusion in HE mice compared to WT mice (Figure 1D). Therefore, the partial deletion of MCT1 blocks the response to high KB concentration sensed by the hypothalamus previously shown to stimulates food intake and body weight gain.

Blood glucose, lactate or KB levels are unaffected as well (Figure 2A,B, and C). However, HE-BHB mice displayed increases of both insulin and leptin levels (Figure 2D,E). The insulin increase combined with euglycemia led to an increased calculated HOMA-IR in HE-BHB mice indicating insulin resistance compared to HE-CSF mice (Figure 2F). Finally, liver, BAT and vsWAT weight are unaffected in HE-BHB mice (Figure 3A,B and C). However, body composition analysis indicated an increased fat mass in HE-BHB mice compared to HE-CSF or WT-BHB mice (Figure 3D).

In addition, HE-BHB mice were significantly more active during the light period (Figure 4E). Indirect calorimetry experiments also revealed an increased RER during the light period in HE-BHB mice (Figure 4D). This result indicates an increased carbohydrate metabolism compared to HE-CSF mice. However, neither VO_2_ nor VCO_2_ showed significant differences which results in similar energy expenditures in both groups (Figure 4A–C).

### 3.6 Expression of hypothalamic neuropeptides and transporters involved in nutrient sensing is altered by a chronic hypothalamic BHB infusion in HE mice

Orexigenic or anorexigenic neuropeptide mRNAs remained unaffected in HE-BHB mice (Figure 5A). Nutrient transporters mRNA expression remained unchanged between groups except for both GLUT3 and GLUT4 which are increased in HE mice compared to the WT littermates (Figure 5B). In addition, there was no observed change in KB metabolism in the hypothalamus (5C).

### 3.7 Peripheral tissues display limited changes in the expression of genes involved in metabolic regulation following chronic hypothalamic BHB infusion in HE mice

Hypothalamic BHB infusion had a marginal effect on liver and vsWAT mRNA expression in HE mice. Moreover, HE-BHB mice showed little changes in BAT and scWAT mRNA expression compared to HE-CSF mice. Interestingly, Cidea and PRDM16 genes involved in browning of scWAT displayed a decrease in mRNA expression levels (Figure 5D). In addition, we also revealed a decrease of PRDM16 in HE mice compared to WT mice (Figure 5D). Further analysis revealed that protein levels of UCP1 in BAT of HE-BHB mice were unaffected (Figure 5E)).

### 3.8 Cold exposure reveals a better tolerance of HE mice chronically infused in the hypothalamus with BHB

Monitoring of the temperature during the 7 days infusion indicated that BHB did not alter body temperature in HE mice (Figure 6A). However, HE-CSF mice exposed to 4 °C were significantly intolerant to cold. On the other hand, BHB infusion provided cold resistance to HE mice when compared to HE-CSF mice as well as compared to WT mice (Figure 6B). Surprisingly, catecholamines levels measured after cold exposure did not differ between HE mice receiving aCSF or BHB, in contrast to epinephrine in WT mice (Figure 6C). In addition, the analysis of UCP1 levels in BAT after cold exposure does not show any changes at the protein level (Figure 6D). Finally, gene analysis in scWAT and BAT only exhibited changes in adrenoceptors mRNA expression levels (Figure 7A–D). Thus, scWAT presented increased mRNA levels of the α1 isoform and decreased mRNA levels of the β3 isoform in HE-BHB mice (Figure 7C). BAT on the other hand does not show any changes (Figure 7D). However, we revealed increased expression of adrenoceptors in HE mice when compared to WT mice in both scWAT and BAT tissues (Figure 7B,D). In parallel, our analysis also revealed an increased expression of the Cidea gene in scWAT and BAT of HE mice when compared to WT mice (Figure 7A,C).

### 3.9 HE mice show marginal differences when compared to WT mice after CSF infusion but opposite effects when receiving BHB

Overall, results obtained recapitulate the previously described phenotype for WT-CSF *vs.* HE-CSF mice. Thus, marginal differences are reported here. Body weight and food intake remain unaffected by the MCT1 deletion. However, RER and locomotor activity are significantly lower in HE mice (Figure 4D,E). At the molecular level, we report here some alterations in tissues involved in energy expenditure regulation. Hypothalamic glucose transporters GLUT3 and GLUT4 are thus increased in HE mice (Figure 5B). In peripheral tissues such as scWAT we also report decreased PRDM16 gene expression while β3 and α1-adrenocaptor expression is increased (Figure 5D). Finally, expression of BAT adrenoceptors β1, β2 and β3 as well as α1 isoforms is shown to be increased in HE mice (Figure 6D) while β3 isoform expression is also increased in scWAT (Figure 6C).

BHB treatment however induces more important alterations in HE mice. Thus, body weight gain and food intake are shown to be decreased by BHB infusion in HE mice comparatively to either CSF-infused WT and HE mice (Figure 1A,B). Similarly, blood leptin is increased in HE-BHB mice compared to the other groups (Figure 2D). This result is paralleled by the fat mass content measured by echoMRI. Finally, Insulin levels follow a similar profile (Figure 2E), which results in an increased HOMA-IR since blood glucose remains unaffected (Figure 2F).

Finally, at the molecular level, hypothalamic GLUT4 expression increases in HE-BHB mice compared to the other groups (Figure 5B). We also observed an increased expression of Cidea in scWAT as well as in BAT tissues (Figure 7A,B). ScWAT from HE-BHB mice also present β1-and α1-adrenoceptor expression increases, whereas β2-, β3-and α1-adrenoceptor expressions are increased in BAT tissue (Figure 7C,D).

## 4 Discussion

The importance of KB in the regulation of metabolism by the brain has generated renewed interest recently [[5], [6], [7], [8], [9], [10]]. Although several studies displayed contradictory results, all agreed that brain detection of KB could participate in obesity onset. In support of such a role, the haploinsufficient mouse model (HE) for the ubiquitous KB transporter MCT1 resists to high fat diet (HFD)-induced obesity [11]. This mouse model does not show any metabolic alteration under a normal chow diet, and KB in the blood are present at low levels. However, during HFD, KB levels increased significantly. Therefore, KB are likely a key element in the resistance to obesity of HE mice that are unable to detect the increased KB levels. Accordingly, we have demonstrated a role of KB in food intake regulation and metabolic imbalance [5,6]. However, while we showed that BHB infusion enhanced food intake, results from other groups reported an inhibition of food intake by KB [[7], [8], [9], [10]]. Although such contradictory results can be explained by differences in methods or models used, we also observed a reverse effect of brain BHB injection on food intake after 12 h, suggesting a multiphasic response to KB [6]. During the first 6 h, high KB could stimulate food intake and thus represent a signal of energy deficit. Under physiological conditions, food ingested should elevate then glucose availability and thus subsequently decrease KB levels.

Previously, we did not observe any alterations between WT and HE mice under a normal chow diet [11]. However, in the current study, a novel analysis revealed some but very limited differences. Of particular interest, we observed various markers involved in energy expenditure control including locomotor activity and thermogenesis. The most striking changes were observed about the expression levels of the Cidea gene in both scWAT and BAT tissues. Such results support an increased thermogenic capacity for both tissues and browning of scWAT. Beigeing of scWAT has previously been linked with protection against obesity development. Thus, this increase could contribute to the diet-induced obesity resistance of this mouse model. Interestingly, such thermogenesis program changes appear as key in the ketone bodies sensing response measured in both WT and HE mice. Altogether, it seems that the MCT1 transporter is a key contributor to thermogenesis mechanism.

### 4.1 Effects in WT mice

Here we provide results in accordance with a biphasic effect of hypothalamic hyperketonemia. Indeed, we observed first an increase in food intake after 24 h of hypothalamic BHB infusion in WT mice. Then, food intake normalized the following 6 days indicating an adaptation to the detected hyperketonemia. These observations confirm and extend our previous results while they reconcile them with the apparently different effect reported by others [7]. Interestingly, HE mice do not show any change in food intake during the 7 days of infusion, supporting a defect of hypothalamic KB sensing due to a reduction of transport in brain cells. However, it is worth noting that HE mice show a decreased food intake compared to WT littermates supporting the role of KB sensing in food intake control. Moreover, these results reinforce the view of a role of hypothalamic KB sensing in food intake control.

Strikingly, body weight of WT-BHB mice is increased, but body composition indicates a decreased lean mass. This increase in body weight paralleled by a decrease in lean mass seems contradictory. One possibility would be that fat is accumulating in this tissue although it is not sufficient to become significant at the level of the whole fat mass analysis obtained by EchoMRI. Therefore, it is likely that a more prolonged hypothalamic BHB infusion would have resulted in a total fat mass increase. Nevertheless, indirect calorimetry analysis showed decreased energy expenditures with a trend for a decreased locomotor activity in WT-BHB mice. These results are in agreement with a reduction in lean mass due to lower physical activity [20]. Moreover, decreased activity and energy expenditures would also result in fat mass accumulation. These results support an obesogenic effect triggered by chronically high KB levels sensed by the hypothalamus. In support of this effect, mice fed with HFD showed an early increase in ketonemia during obesity development [14]. This increased ketonemia is induced by HFD that provides large amounts of lipids. In turn, liver metabolism is responsible of ketogenesis [21]. The resulting high KB concentration will then cause a decrease in physical activity and energy expenditure that would contribute to fat storage and loss of lean mass. Furthermore, the observed hyperinsulinemia in WT-BHB mice will also contribute to stimulate fat accumulation by stimulating lipogenesis [22].

High insulin levels associated with euglycemia suggests insulin resistance as reported by the calculated HOMA-IR. This result is consistent with previous ones indicating muscle insulin resistance induced by KB [23,24]. The decreased physical activity could participate to the development of insulin resistance since it is well established that physical activity and insulin sensitivity are tightly linked to each other [[25], [26], [27]]. Indeed, an increase in physical activity improves insulin sensitivity through increased GLUT4 expression at the cellular membrane [17,28]. Therefore, a reduction of physical activity would be associated to a lower GLUT4 localization at the membrane and thus a lower insulin sensitivity. Our results indicate that cerebral KB sensing influences peripheral insulin sensitivity. In addition, since blood KB levels were unchanged, it is likely that a central signal to the periphery would be responsible for such insulin resistance. However, the nature of the signal involved remains to be determined.

Furthermore, the mechanism causing the reduction in physical activity remains to be determined. As BHB was injected in the hypothalamus, a direct effect on the motor cortex can be excluded. Among the different hypothalamo-cortical connections, the control of the arousal response is most likely to impact physical activity. In support of this view, VMH neurons have been linked to arousal regulation in previous work [29,30]. Therefore, future exploration of this pathway will be necessary to better understand the role of brain ketone bodies sensing in the regulation of physical activity.

Alterations of hypothalamic regulatory mechanisms are supported by changes in the levels of hypothalamic neuropeptides described previously following KB stimulation [5,6]. Although hypothalamic neuropeptides are not affected, a role of NPY neurons in KB sensing is possible since 50% of these neurons colocalize with MCT1 and are expected to be direct sensors of KB levels [5]. Beside stimulating food intake, NPY is known to reduce energy expenditures as we observed in our study [31]. KB transport in NPY neurons would represent a signal of energy deficiency that would trigger a counter-regulatory response to increase energy supply, although we can not exclude a contribution of other MCT1-expressing brain cells to KB sensing. Indeed, the trend toward a decreased MCT4 expression indicates a lower lactate efflux from astrocytes [32]. This result would suggest a decreased supply of energy substrates from astrocytes to neighboring neurons. Indeed, glucose metabolism in astrocytes leads to increased lactate production that is in turn transferred to neurons to sustain their energy needs via MCT4 in astrocytes and MCT2 in neurons. Such a decrease in astrocytic MCT4 expression suggests that KB could have an impact by reducing the metabolism of astrocytes. One possible explanation for such an effect could come from increased glucose uptake by neurons. By reducing their lactate use, it would limit glucose uptake in astrocytes and contribute to reduce glycolysis and lactate production, resulting in decreased MCT4 expression. However, the existence of such a mechanism remains to be tested.

Hypothalamic infusion of BHB for 7 days led to gene expression changes in scWAT and BAT while similar liver or vsWAT gene expressions were unaffected. In addition, BAT also presented decreased UCP1 protein levels that should be associated with decreased thermogenesis [33]. Overall, these results seem to contradict the accumulation of lipids in hypothalamic BHB-infused mice. However, cold exposure showed that WT-BHB mice display a cold tolerance not different from control mice receiving aCSF. This result suggests that thermogenesis in both mice is similar despite the alterations in some important metabolic gene expression levels. This is supported by a similar level of UCP1 protein expression in BAT. An effective hyperactivation of the sympathetic nervous system by cold exposure is evidenced by the increased epinephrine concentration measured in WT-BHB mice. Epinephrine is responsible for the increased activity in BAT cells to generate heat and participate to thermogenesis [34].

Most interestingly, expression analysis of genes known to be involved in the browning of WAT showed that both Cidea and PRDM16 mRNA expressions were decreased. These genes have been demonstrated to participate in a metabolic adaptation of scWAT. Their increased expression is associated with increased browning of WAT cells and a decreased risk of obesity development. On the other hand, decreased expression of these markers leads to an increased risk of obesity [35]. Therefore, in our study we demonstrated that brain detection of KB drives a limited browning capacity of scWAT. Consequently, WT-BHB mice display an increased risk of obesity development consistent with the overall phenotype observed.

### 4.2 Effects in HE mice

Altogether, our results support the view that high KB levels sensed by the brain increase the risk of obesity by altering peripheral metabolism. These results also suggest that part of the resistance to obesity development of HE mice results from a reduced central KB sensing. Indeed, MCT1 represents a major transporter for KB expressed notably by endothelial cells [36]. Therefore, it favors the transport of KB within brain structures. In parallel, our previous work supports a role of NPY neurons in KB sensing that is in accordance with the phenotype observed in wildtype mice. These observations led us to test the effect of direct BHB infusion to the brain of HE mice and repeat the same experiments than in WT mice.

Very interestingly, we noticed that HE mice not only do not recapitulate the phenotype observed in WT mice when infused with BHB, but rather showed a phenotype that is in favor of a decreased risk of obesity.

Food intake and body weight of HE mice were not altered by hypothalamic BHB infusion. Furthermore, we measured a decrease in food intake compared to WT mice supporting a role of KB sensing in food intake control. In addition, leptin levels were significantly increased. This increase parallels the significant fat mass increase detected and the elevated insulin levels that should contribute to the leptin increase observed. The inhibitory role of leptin on food intake could be responsible for the disappearance of food intake stimulation and body weight gain increase observed in WT mice [37]. Insulin levels, however, remained elevated in mice infused with BHB and resulted in an elevated HOMA-IR, indicating that insulin resistance is still present in these mice. This result could be due to KB sensing by a specific cell population not dependent on MCT1 expression. Indeed, other MCTs are expressed by neurons, and in particular MCT2 which is the major neuronal MCT in the CNS [36]. It is thus possible that other neuronal populations triggered the insulin resistance phenotype. However, this hypothesis would need to be tested directly at the electrophysiological level in the hypothalamus. This experiment could allow to identify some hypothalamic neuronal populations in HE mice that would show decreased activity in presence of high levels of BHB. Indeed, the observed increased expression in GLUT transporters in HE mice compared to WT mice suggest altered nutrient fluxes in the hypothalamic area and could thus have effects in various neuronal populations.

HE mice do exhibit a response to hypothalamic BHB infusion that can be detected by performing peripheral analyses. Since the expression of MCT1 is decreased by 50% in the brain [11], it is expected that the signal of high circulating BHB is still sensed, although the decreased in MCT1 expression would results in a lower intracerebral flux of BHB. One possible explanation of such a partial effect could be that the amount of sensed BHB would result in different signals. A high level of BHB would induce deleterious responses, while lower BHB levels, although higher than normal, would have a positive effect.

Among the changes induced by central KB infusion, AMPK has been shown to be inhibited in the hypothalamus, an observation consistent with the inhibition of food intake [38,39]. Furthermore, this result is also consistent with previous results indicating an inhibitory effect of KB on food intake [[7], [8], [9], [10]]. Despite being in contrast with our previous studies, this result supports the hypothesis of a low dose effect here. Indeed, in other studies showing the inhibitory effect of KB on food intake, the authors’ strategy to induce KB production relied on a high fat diet. Such a diet induces an increased ketonemia but to a lesser extent than achieved with the infused dose we used in our studies [14].

Results obtained from peripheral tissue measurements in HE mice are partially similar to the results in WT mice. However, a striking increased in Cidea levels in BAT from HE mice is also revealed compared to the WT mice. This result strongly indicates a thermogenic role of MCT1 in BAT that would need deep exploration. Nevertheless, gene expression levels support a shift in fat cells metabolism induced by BHB infusion in the hypothalamus. Thereby, gene expression in HE-BHB mice suggest a similar role of brain KB sensing in thermogenesis and browning. However, we observed a significant increase in fat mass. BHB infusion could have led to a decrease in fat utilization rather than fat accumulation. This is supported by the increased RER that indicates an increased oxidation of carbohydrates instead of lipids. This shift in metabolic oxidation would then preserve fat stores. In addition, calorimetric studies also indicate an increase in energy expenditures that seems to be due to increased activity.

We also describe a better tolerance to cold exposure of HE-BHB mice. Such an effect on thermogenesis would contribute to the resistance to diet-induced obesity, by participating to the increased energy expenditures. This is supported by a higher UCP1 protein expression in BAT of HE-BHB mice. Here, in contrast to WT mice, the decreased browning capacity suggested does not impact thermogenic mechanisms. This result suggests that although the MCT1 transporter expression is decreased, HE mice can still sense variations in BHB levels. However, in this case, the signal appears to be beneficial and induces adaptations that ultimately protect against obesity development.

### 4.3 Conclusions

Altogether, our results support the existence of a differential response to a low *vs.* a high concentration of circulating KB such as BHB. Such a hypothesis will require to be tested in mice to clearly determine the threshold between the beneficial and deleterious effects. In accordance, several reports indicate that KB are involved in obesity development, although mechanisms are still unclear [7]. On the other hand, ketogenic diets are used in weight loss management successfully [40]. Moreover, ketogenic diets are also often used in the treatment of some juvenile forms of epilepsy or other neurological diseases with beneficial effects [41]. Interestingly, among the mechanisms suspected to be implicated, effects on inflammation, redox regulation, mitochondrial function and ATP production or even neuronal plasticity are also described as involved in brain nutrient sensing and energy homeostasis regulation [41].

Overall, BHB appears to have dual effects in the different models we used in this study. When the transporters of KB are fully functional, brain sensing of the hyperketonemia produces deleterious effects. However, when the transport of BHB is reduced, BHB produces a beneficial effect that contributes to obesity resistance. Nevertheless, both involve a brain to periphery regulation of tissues involved in energy homeostasis. Of particular interest, browning of subcutaneous WAT is affected in both cases. Furthermore, the striking increased of Cidea expression in BAT from HE mice supports a role for MCT1 in thermogenesis. Also, in support of this WAT metabolism regulation, cold tolerance is also involved in the KB sensing response. Finally, the dual effect of BHB on metabolic regulation as described here remains to be fully understood. Notwithstanding, our results open new perspectives about the mechanisms of both obesity development and obesity resistance.

## CRediT authorship contribution statement

**Lionel Carneiro:** Writing – review & editing, Writing – original draft, Visualization, Validation, Supervision, Software, Resources, Project administration, Methodology, Investigation, Funding acquisition, Formal analysis, Data curation, Conceptualization. **Rocco Bernasconi:** Formal analysis, Data curation. **Adriano Bernini:** Formal analysis, Data curation. **Cendrine Repond:** Formal analysis, Data curation. **Luc Pellerin:** Writing – review & editing, Writing – original draft, Validation, Supervision, Resources, Project administration, Investigation, Funding acquisition, Conceptualization.

## Declaration of competing interest

The authors declare that they have no known competing financial interests or personal relationships that could have appeared to influence the work reported in this paper.

## Data Availability

Data will be made available on request.
