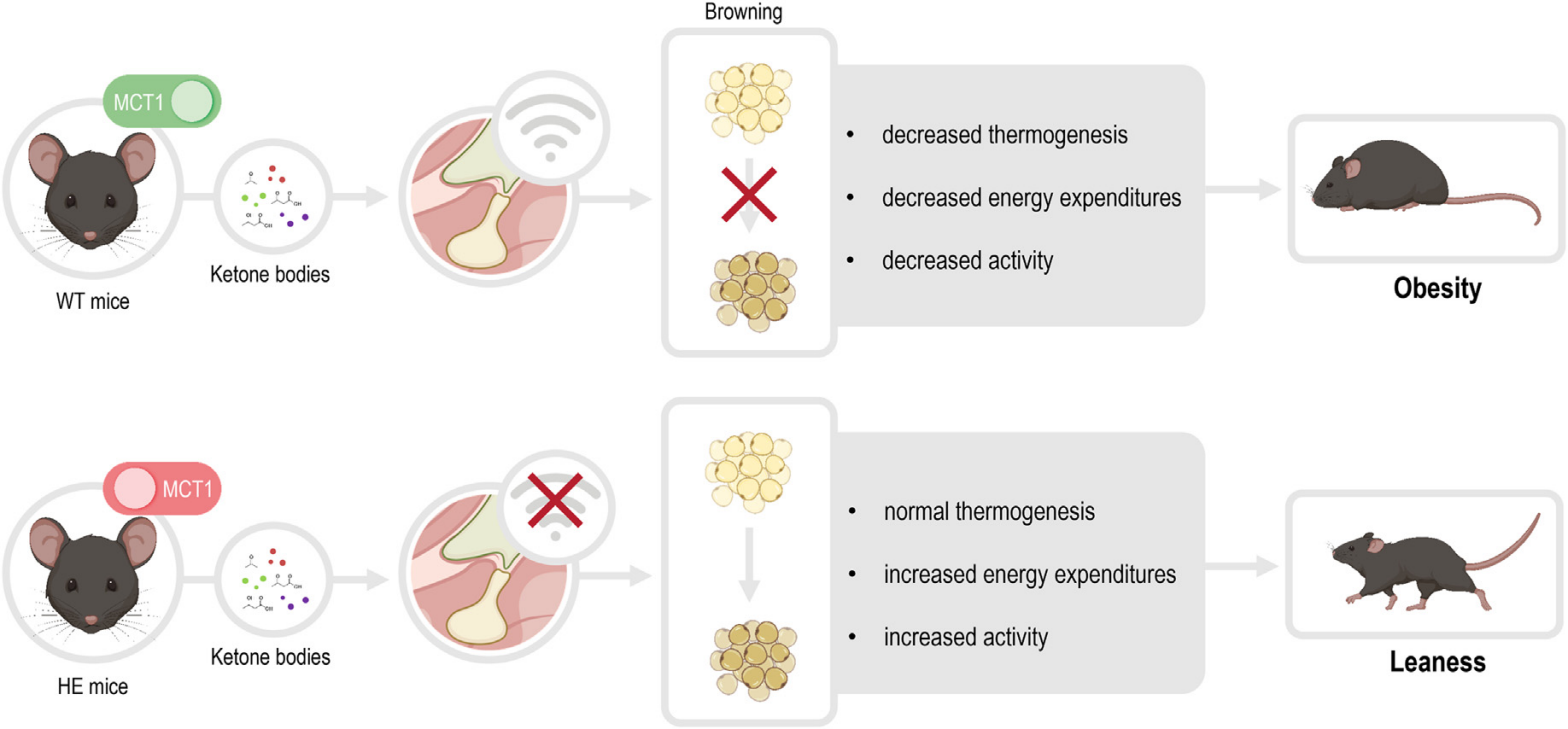# Corrigendum to “Elevation of hypothalamic ketone bodies induces a decrease in energy expenditures and an increase risk of metabolic disorder” [Mol Metab, 83 (2024) 101926]

**DOI:** 10.1016/j.molmet.2024.101937

**Published:** 2024-04-12

**Authors:** Lionel Carneiro, Rocco Bernasconi, Adriano Bernini, Cendrine Repond, Luc Pellerin

**Affiliations:** 1 Department of Physiology, University of Lausanne, 1005 Lausanne, Switzerland; 2 University and CHU of Poitiers, INSERM, U1313, Poitiers, France

The authors regret to have forgotten the submission of a graphical abstract along the main manuscript. The graphical abstract is now added in this corrigendum.

The authors would like to apologise for any inconvenience caused.

**Figure:**